# Evaluating orange oil and lespedeza hay as an alternative anthelmintic for goats

**DOI:** 10.1093/tas/txaf083

**Published:** 2025-06-21

**Authors:** Nicolas Caram, Emanuel Duvalsaint, Diwakar Vyas, Marcelo Wallau

**Affiliations:** Agronomy Department, University of Florida, Gainesville, FL 32611, USA; Agronomy Department, University of Florida, Gainesville, FL 32611, USA; Department of Animal Sciences, University of Florida, Gainesville, FL 32611, USA; Agronomy Department, University of Florida, Gainesville, FL 32611, USA

**Keywords:** anthelmintic, *Haemonchus contortus*, lespedeza, orange oil

## Abstract

Identifying alternative practices to control gastrointestinal parasites and overcome resistance to chemical anthelmintics is critical to maintain the productive and economic viability of the small ruminant sector. Here, we evaluated in vivo potential effects of orange oil and sericea lespedeza (*Lespedeza cuneata* [Dumont] G. Don) supplementation on suppressing gastrointestinal nematodes (GIN) and improving performance of goats. Twenty-four bucks were used in a randomized complete block design and were assigned to12 pens after deworming. Experimental duration was 6 wk (1 wk adaptation and 5 wk of data collection) and treatments were arranged in a factorial combination of two levels of orange oil, at 0 or 600 mg/kg BW, and lespedeza hay, at 0% or 9% of the diet DM, in three replicates. Response variables included dry matter intake (DMI), growth performance, nutrient digestibility, fecal egg counts (FEC), and blood parameters. The inclusion of orange oil and/or lespedeza decreased DM (−12.6%) and neutral detergent fiber digestibility (−14.3%) but did not decrease DMI (1.06 kg DM/goat/day) or growth performance (78.3 g/animal/day). Orange oil administration decreased 46% of Trichostrongyle FEC, but no effects were observed on strongyloide and coccidia FECs. Orange oil also increased neutrophil concentration, potentially indicating an inflammatory response. Although orange oil showed promise as a natural alternative to synthetic dewormers for controlling GIN in goats, its efficacy is variable and may be influenced by dosage, formulation, and treatment duration. However, lespedeza hay, despite its known antiparasitic effects, was ineffective in reducing FEC when included at levels in this experiment.

## Teaser

Our research has demonstrated a 46% reduction in Trichostrongyle fecal egg counts with orange oil supplementation without compromising growth performance. Findings highlight the potential of plant-based anthelmintics in sustainable small ruminant production systems.

## INTRODUCTION

Sheep and goat production has recently gained popularity in the U.S. and is viewed as an emerging sector that provides local, grass-fed, and organic meat to alternative markets (Terrill et al., 2009). However, management of gastrointestinal parasites (e.g., *Haemonchus* spp., *Trichostrongylus* spp., *Teladorsagia* spp., *Cooperia* spp. *Nematodirus* spp. and *Oesophagostomum* spp) has been one of the major challenges for small ruminant producers in the humid Southeast (Bishop, 2012), limiting productive and economic outcomes (Scott and Sutherland, 2009; Sadiqqi et al., 2011). Synthetic anthelmintics are the most common method to prevent and control gastrointestinal parasites (Claerebout et al., 2020; Mphahlele et al., 2021). However, throughout the last few decades, the frequent overuse of anthelminthic drugs has resulted in parasite resistance (Githiori et al., 2004). Additionally, these drugs have been identified as a public health issue due to their metabolic residues in animal meat and milk if not administered well (Abbas et al., 2020).

Recently, there has been an increased interest in alternative strategies and natural products that can help with gastrointestinal nematode (GIN) management in small ruminants (Ferreira et al., 2016; Katii et al., 2017; Macedo et al., 2019; André et al., 2018; Zaman et al., 2020; Štrbac et al., 2022). Among the most concerning parasites is *Haemonchus contortus*, a blood-feeding GIN that thrives in warm and humid environments, causing anemia and low blood cell levels in the host, compromising animal welfare and potentially leading to death (Whittier et al., 2009; Saddiqi et al., 2011). Plant-based bioactive compounds of terpenes (essential oils) and other secondary metabolites, such as tannins and alkaloids, have been proposed as natural anthelmintics (Ramdani et al., 2023). Essential oils are bioactive compounds composed of aromatic terpenoids, aliphatic components, and terpenes (Squires et al., 2010; Zhu et al., 2013 ; Fayaz et al., 2019; Helal et al., 2020). Essential oils from *Eucalyptus citriodora* and *E. staigeriana* effectively controlled *Haemonchus contortus* in sheep (Macedo et al., 2019 ; Araújo-Filho et al., 2018), with improved efficacy over ivermectin (61% vs. 49%; de Aquino Mesquita et al. 2013) and were shown to decreased fecal egg count (FEC) between 61% to 77% in goats (Macedo et al., 2010; Mesquita et al., 2013). Essential oils from citrus decreased the fecal egg count in 97.4% in small ruminants dosed with orange oil emulsion containing 95% limonene (Squires et al., 2010).

Other secondary plant metabolites such as condensed tannins and other polyphenolic compounds (Mangan 1988) were shown to decrease FEC (Hoste et al., 2006; Villalba et al., 2012; MacAdam and Villalba, 2015) without compromising growth performance in small ruminants (Min et al., 2004) when added at to the diet at moderate levels (<8%; Hoste et al. 2006). Heckendorn et al. (2006) and Villalba et al. (2012) reported a 58, 48 and 20% decrease in FEC when feeding sainfoin to lambs infected with *H. contortus*, and Heckendorn et al. (2007) reported a 63% decrease in FEC in lambs fed birdsfoot trefoil (1% to 4% tannin). In the south, sericea lespedeza (*Lespedeza cuneata* [Dumont] G. Don) (4% to 22% condensed tannins; Windham et al., 1988; Shaik et al., 2004; Puchala et al., 2016 ) has been promoted as having anthelmintic proprieties (Min et al., 2004; Shaik et al., 2004;  Terrill et al. 2009; Mechineni et al., 2014). Terrill et al. (2009) reported between 84.6% and 91.9% reduction in FEC in goats fed 50% to 75% of the diet as sericea lespedeza, compared to bermudagrass hay for 41 d, although intake and performance were not measured. This species, however, is considered invasive in western and certain southern states in the US and not recommended for planting in those regions.

Alternative approaches are needed to control gastrointestinal parasites, overcome resistance to chemical anthelmintics, and maintain the productive and economic viability of the small ruminant sector in tropical and subtropical environments. Practices such as supplementation with essential oils and tannins, along with pasture rotation and physical disturbance (e.g., mowing or tillage), are among the proposed strategies used for parasite management (Nguyen et al., 2005; Ferreira et al., 2016; Katiki et al., 2017; André et al., 2018; Zaman et al., 2020; Štrbac et al., 2022 ). In this study, our objective was to evaluate in vivo potential effects of orange oil and sericea lespedeza supplementation on suppressing gastrointestinal parasites and improving the growth performance of goats. We hypothesized that combining both treatments could reduce FEC in goats and enhance the average daily gain through improved nutrient utilization.

## MATERIALS AND METHODS

The experiment was conducted at the University of Florida’s Sheep Unit in Gainesville, Florida, between November and December 2021. The University of Florida Institutional Animal Care and Use Committee approved all experimental procedures involving animals.

### Animals, Treatments, and Experimental Design

Twenty-four bucks, Kiko and Boer; (4 to 6 mo; 16 ± 9 kg), were blocked with three levels of fecal egg count (low, medium, high) and distributed based on weight to 12 experimental pens (2 animals per pen) in a complete randomized block design with three replicates. The pens (1.7 × 7.3 m) were in an open barn under a protective roof. Animals were provided with water ad libitum throughout the study period. Prior to the trial, animals were kept in an adjacent barn and fed with bermudagrass with a commercial “Premix” (Table 1) for 28 d for acclimation.

**Table 1.** Chemical composition of bermudagrass, lespedeza and Premix^1^ that composed the experimental diets. Chemical composition includes dry matter (DM), organic matter (OM), crude protein (CP), neutral (NDF) and acid detergent fiber (ADF) and acid detergent lignin (ADL) concentration (± SD)

	Bermudagrass	Lespedeza	Premix^1^
DM (%)	93.1 ± 0.1	92.8 ± 0.1	91.6 ± 0.1
OM (%)	93.0 ± 0.3	91.7 ± 0.2	94.1 ± 0.1
CP (%)	10.6 ± 0.3	13.4 ± 0.4	11.9 ± 0.6
NDF (%)	66.0 ± 0.1	56.2 ± 0.2	21.6 ± 0.9
ADF (%)	35.4 ± 0.1	44.0 ± 0.1	13.1 ± 0.3
ADL (%)	11.1 ± 2.0	22.6 ± 0.7	2.9 ± 0.1

^1^Premix is composed of alfalfa pellets and meals, cracked corn, cornmeal, cottonseed, cottonseed hulls, oats, soybean meal, soyhull pellets, Bovatec®91 ionophore (Zoetis, Kalamazoo, MI), molasses, VitaFerm mineral mix (VitaFerm, St. Joseph, MO).

Treatments were a factorial combination of two levels of orange oil, at 0 or 600 mg/kg BW, and lespedeza hay, at 0% or 20% of the diet dry matter (DM). Orange oil was dosed orally at the beginning of the trial. Basal diet was constituted of ‘Coastal’ bermudagrass hay (40%) and premix concentrate (60%). For treatments including lespedeza, 8.7% of bermudagrass hay was substituted on a DM basis. Animals were fed ad libitum based on Nutrient Requirements for Small Ruminants (NRC, 007), for an average weight of 20 kg with a target gain of 100 g/day (NRC, 2007) . The chemical composition of the experimental diets is presented in Table 2. Experimental animals were fed at 0700 a.m daily. The quantity of feed offered to each pen was recorded at each feeding time and amounts offered were adjusted to keep 5% refusals. Orts were collected and weighed for each pen once daily before morning feeding.

**Table 2.** Ingredient and nutrient composition (± SD) of bermudagrass and bermudagrass + lespedeza experimental diets. Nutrients composition include dry matter as fed (DM), organic matter (OM), crude protein (CP), neutral (NDF) and acid detergent fiber (NDF), and acid detergent lignin (ADL) concentration

	Experimental diets	
Ingredients, % of diet DM	Control (Bermudagrass)	Lespedeza (Bermudagrass + Lespedeza)
Bermudagrass hay	33.8	25.7
Lespedeza hay	0	8.7
Molasses	6.3	6.3
Grain premix	74.0	74.6
Alfalfa pellets	2.8	2.8
Alfalfa meal	5.7	5.7
Corn, cracked dry	39.6	39.6
Distillers grain with solubles	19.2	19.2
Cottonseed hulls	8.5	8.5
Oats, rolled	8.5	8.5
Soybean meal, 48% CP	10.2	10.2
Soybean hulls	4.0	4.0
Bovatec-91^1^	0.01	0.01
Molasses	9.1	9.1
Vitaferm^2^	5.7	5.7
Nutrient composition, % DM		
DM % as-fed	87.7 ± 0.8	87.6 ± 0.8
OM %	94.1 ± 0.3	93.6 ± 0.1
CP %	11.9 ± 0.1	11.6 ± 0.2
NDF %	37.4 ± 3.2	41.8 ± 2.9
ADF %	20.9 ± 1.7	24.7 ± 1.9
ADL %	4.4 ± 0.4	6.8 ± 0.8

^1^Zoetis, Kalamazoo, MI.

^2^VitaFerm, St. Joseph, MO.

Orange oils were products of Florida Chemical Co. Inc. (Winter Haven, FL, USA). The experimental composition of the orange oil emulsion used in this study contained 60% orange terpene oil, 4% polysorbate 80, 1.5% hydrogen peroxide, and 34.5% water, as described and evaluated by Squires et al. (2010).

### Timeline of Experiment

Experimental animals were dewormed with a combination of fenbendazole (5 mg/Kg), levamisole (8 mg/kg), and moxidectin (0.2 mg/kg) 4 wk before the initiation of the adaptation period. To assess the effectiveness of this treatment, fecal samples were taken at 2- and 4-wk post-deworming. Based on the fecal egg count results from these samples, animals were blocked into low, medium, and high FEC groups. The experimental diets were introduced at the onset of the adaptation period, which commenced 4 wk following the deworming process. A week into the adaptation period and at the start of the 5-wk experimental phase, orange oil was dosed orally to animals in the pens receiving orange oil treatment and the other half received placebo (same emulsion without orange oil). Fecal samples were collected on days 0, 1, 2, 6, 7, 8, 14, and 21 following the orange oil treatment. During week 3 and 5, fecal samples were collected to estimate apparent nutrient digestibility.

### Response Variables

#### Feed intake and nutritive value.

Representative samples of the feed and orts were collected and dried at 55 °C for 48-hours in a forced-air oven to estimate DM concentration, then ground in a Wiley mill using a 1-mm screen (A. H. Thomas Co., Philadelphia, PA). Daily feed intake was calculated as the difference between feed offered and orts, expressed on a DM basis. Dry matter intake (DMI) was considered a weekly average feed intake. Samples of diets, offered refusals, and individual feed ingredients were collected and stored for further analysis.

Total mixed ration and orts were analyzed for dry matter (DM), crude protein (CP), neutral detergent fiber (NDF), acid detergent fiber (ADF), and ash content. The ash content was determined by incinerating the sample in a muffle furnace at 600 °C overnight and then weighing the remaining residue (method 942.05, AOAC International, 1990). Crude protein concentration was analyzed at the Forage Laboratory of the Agronomy Department of the University of Florida, Mariana, FL). The N concentration was determined by rapid combustion using a micro elemental N analyzer (Vario Micro Cube, Elementar Analysensysteme GmbH), following AOAC International method 992.15 (AOAC International, 1995  ), and CP values were calculated by multiplying N concentrations by 6.25. Neutral detergent fiber (sodium sulfite and alpha-amylase; NDF; method 2002.04; AOAC International 2000) and acid detergent fiber (method 7.074; AOAC International 2000) were analyzed sequentially using a fiber analyzer (200/20, Ankom Technology).

#### Average daily gain.

Body weight was recorded weekly throughout the experiment using a scale (LBS INC. weigh cage with an Avery Weigh-Tronix ZM301 indicator) before morning feeding to estimate average daily gain (g/day). The average daily gain was calculated weekly as the difference between the final and initial weights of each pen, divided by 2 animals in the pen, by 7 d. Body condition grading, which subjectively categorizes animals into five scores [from thin (1) to obese (5)] based on the quantity of subcutaneous fat deposits over particular bony protuberances, was also assigned weekly for each animal  (Van Saun et al., 2009). 

#### FAMACHA and body condition score.

We estimated the FAMACHA score primarily to assess the severity of anemia in experimental animals and as a management practice to control parasite infection. To measure the FAMACHA score, a handler restrained the animal while the scorer gently pulled down the lower eyelid to reveal the color of the mucous membrane. The observed color was then compared to the FAMACHA card to assign a score on a scale of 1 (not anemic; ideal) through 5 (severely anemic; Storey et al. 2017).

#### Fecal sampling.

Fresh fecal samples were collected at day 0, 1, 2, 6, 7, 8, 14, and 21, through rectal palpation from each animal for determination of FEC (eggs per g); using the modified McMaster’s method (Whitlock, 1948) . For this analysis, 28 mL of sodium chloride solution was mixed with 2 g of fecal matter and stirred until dissolved. The mixture was then filtered to remove large particles to ensure that only the eggs and the solution entered the counting chamber of the McMaster slide. Each chamber of the McMaster slide was filled using a plastic transfer pipette with the fecal solution. On a 10X objective, slides were read, and eggs of different species were identified. The total number of eggs for each species was multiplied by 50 to determine eggs per gram.

#### Blood collection.

Blood was collected weekly from the jugular vein of each animal using an EDTA Vacutainer tube (3 ml). Samples were analyzed for complete blood counts, such as red blood cells, white blood cells, and hemoglobin using the IDEXX ProCyte DX analyzer with the appropriate species setting described by Herndon et al. (2020).

#### Apparent digestibility.

Diet digestibility was evaluated during week 3 and 5 of the experiment. Diet samples were collected for 3 d. Spot fecal samples were collected during week 3 and 5 (4 samples/pen) for 3 d at 0600, 1000, 1400, and 1800 on day 1; 0700, 1100, 1500, and 1900 on day 2; and 0800, 1200, 1600, and 2000 on day 3. Samples were composited, dried in a forced-air oven for seven days at 55 °C, and ground to pass through the 1 mm screen of Wiley mill (A. H. Thomas, Philadelphia) and analyzed for absolute DM (105 °C overnight; AOAC, 1990; method 930.15) and organic matter (OM) estimation (AOAC, 1990; method 942.05). Acid detergent lignin (ADL) was used as an internal marker to estimate fecal output. ADL in ort and feces was analyzed according to the batch procedures outlined by ANKOM Technology Corp. (Fairport, NY, USA). Diet samples, refusal, and fecal samples were incubated in 72% sulfuric acid in an Ankom Daisy incubator jar. Fecal output (kg DM/d) was calculated as,


$$\begin{array}{l} Fecal\;output\left(kg\;DM/d\right)=\frac{ADL\;intake\left(kg\;DM/d\right)}{ADL\;\%\;\;feces} \end{array}$$ (1)

where the fecal output was estimated as the ratio between ADL intake and ADL concentration in feces (%). The intake of ADL (kg DM/d) was estimated as:


$$\begin{array}{lll} ADL\;intake\left(kg\;DM/d\right)= & \;\left(ADL\;\%\;\;diet\times diet\;of\;fered\right)--\; & \;\left(ADL\;\%\;\;refusal\times refusal\right) \end{array}$$ (2)

where ADL intake (kg DM/d) is the difference between the content of ADL offered and ADL refused.

Apparent digestibility (%) was estimated for DM, OM, NDF, and ADF, as:


$$\begin{array}{l} Ap\;dig\left(\;\%\;\right)=100\text{–}\frac{Nutrient\;\%\;\;feces\times fecal\;output\left(kg\;DM/d\right)}{Nutrient\;intake\;(kg/d)} \end{array}$$ (3)

where, nutrient content in feces is estimated as the nutrient concentration and the fecal output, and nutrient intake (kg DM/d) is estimated as:


$$\begin{array}{lll} Intake\left(kg\;DM/d\right)= & \;\left(nutrient\;\%\;\;diet\times diet\;of\;fered\right)\; & \text{–}\left(nutrient\;\%\;\;refusal\times refusal\right) \end{array}$$ (4)

where intake is the difference between nutrient content in the diet offered and nutrient content in refusal.

### Statistical Analysis

Data analysis was performed using the PROC GLIMMIX procedure of SAS (SAS Institute, Cary, NC). The effect of orange oil, lespedeza supplementation, sampling date, and the two-way and three-way interactions on diet digestibility were assessed using a linear mixed model, as:


$$\begin{array}{lll} y_{ijkl}= & \;\mu +O_{i}+L_{j}+OL_{ij}+D_{k}+DO_{ik}\; & +DL_{jk}+DOL_{ijk}+r_{l}+iBW_{ijkl}+\varepsilon_{ijkl} \end{array}$$

where, *y* is the response variable, O is the effect of *i*^*th*^ orange supplementation, L is the effect of *j*^*th*^ lespedeza supplementation, D is the *k*^*th*^ sampling week, *r* is the *l*^*th*^ block effect and BW is the covariate effect of body weight at *ijkl* pen. The effects of orange, lespedeza, sampling week, and interactions were included as fixed effect, while block was included as a random effect. The correlation in time between sampling dates was included using autoregressive correlation [AR(1)].

The effect of orange oil, lespedeza, and week effect on DMI (kg animal/day, % BW, and g BW^0.75^), body condition score, and FAMACHA were assessed performing linear mixed models, as:


$$\begin{array}{lll} y_{ijkl}= & \;\mu +O_{i}+L_{j}+OL_{ij}+W_{k}+OW_{ik}\; & +LW_{jk}+OLW_{ijk}+r_{l}+iBW_{ijkl}+\varepsilon_{ijkl} \end{array}$$

where, *y* is the response variable, O is the effect of *i*^*th*^ orange supplementation, L is the effect of *j*^*th*^ lespedeza supplementation, W is the *k*^*th*^ week effect, *r* is the *l*^*th*^ block effect, and iBW is the covariate effect of initial BW of *ijkl* pen. Orange, Lespedeza and Week were included as fixed effects, while block was included as random effect. An autoregressive correlation [AR(1)] was chosen to account for the dependence in time.

The average daily gain for the entire period was assessed including orange oil and lespedeza as fixed effects and block as random effect, as:


$$\begin{array}{l} y_{ijk}=\mu +O_{i}+L_{j}+OL_{ij}+r_{k}+iBW_{ijk}+\varepsilon_{ijk} \end{array}$$

where, *y* is the average daily gain, O is the effect of *i*^*th*^ orange supplementation, L is the effect of *j*^*th*^ lespedeza supplementation, *r* is the *l*^*th*^ block effect and iBW is the covariate effect of initial body weight at *ijk* pen.

The effect of orange oil and lespedeza on fecal egg count of Trichostrongyle, Strongyloide and Coccidia during the 21-d period was assessed performing a linear mixed model, where orange oil, lespedeza, sampling day and two- and three-way interaction were included as fixed effects, while block was included as random effect, as:


$$\begin{array}{lll} y_{ijkl}= & \;\mu +O_{i}+L_{j}+OL_{ij}+D_{k}+OD_{ik}\; & +LD_{jk}+OLD_{ijk}+r_{l}+\varepsilon_{ijkl} \end{array}$$

where, *y* is the response variable, O is the effect of *i* orange supplementation, L is the effect of *j*^*th*^ lespedeza supplementation, D is the *k*^*th*^ day effect and *r* is the *l*^*th*^ block effect. The correlation in time was modeled as an unstructured correlation (UN).

Lastly, the treatment effects on blood parameters was assessed as:


$$\begin{array}{lll} y_{ijkl}= & \;\mu +O_{i}+L_{j}+OL_{ij}+W_{k}+OW_{ik}\; & +LW_{jk}+OLW_{ijk}+r_{l}+\varepsilon_{ijkl} \end{array}$$

where, *y* is the response variable, O is the effect of *i*^*th*^ orange supplementation, L is the effect of *j*^*th*^ lespedeza supplementation, W is the *k*^*th*^ week effect and *r* is the *l*^*th*^ block effect. The correlation in time was modeled as an autoregressive correlation of first order [AR(1)]. All models were assessed graphically following the suggestions of Kozak and Piepho (2017) .

## RESULTS

### Digestibility and Animal Responses

Dry matter and OM digestibility was decreased with the inclusion of orange oil (*P* = 0.03) or lespedeza (*P* = 0.02; Table 3). Similarly, NDF digestibility tended to decrease with orange oil dosing and feeding with lespedeza (*P* = 0.07; −14.3%) compared with the ‘control’ diet. The ADF digestibility tended to decrease when goats were dosed with orange oil (*P* = 0.07) or supplemented with lespedeza (*P* = 0.05). However, no treatment effects were observed on DMI with the inclusion of orange oil and/or lespedeza in diet (Table 3). The daily DMI across all treatments averaged 1.06 kg/animal, equivalent to 4.3% of the BW and 95.7 g DM BW^0.75^. Overall, goats increased 20% of the daily DMI from week 1 to week 5 (0.95 to 1.15 kg DM/day; *P* < 0.01), 21% of the daily intake expressed as % of BW (3.9 to 4.7% of BW; *P* < 0.01) and 20% in intake related to metabolic body weight (86.1 to 103.7 g DM BW^0.75^; *P* < 0.01).

**Table 3.** Treatment effects on digestibility of dry matter (DMD), organic matter (OMD), neutral detergent fiber (NDFD) and acid detergent fiber (ADFD), on dry matter intake (DMI) in kg/animal, % body weight (BW) and in g/metabolic body weight (BW^0.75^), and on body condition score (BCS), FAMACHA, and animal performance (g/day) in response to Orange oil (O) administration and inclusion of Lespedeza (L) hay in the total mixed ration. *P* values only show the effect of orange oil, lespedeza, time, and the two-way interaction between orange oil and lespedeza (O × L)

	Treatments					*P* values			
	No Lespedeza		With Lespedeza						
	Control	Orange	Lespedeza	O + L^3^	SE	Orange	Lespedeza	O × L	Time^1^
DMD (%)	74.0	70.9	70.0	61.0	2.3	0.03	0.02	0.21	0.09
OMD (%)	75.4	72.6	71.3	62.8	2.1	0.03	0.02	0.19	0.09
NDFD (%)	51.1	49.8	50.9	36.2	4.1	0.04	0.06	0.07	0.74
ADFD (%)	48.9	47.7	46.9	35.2	4.3	0.07	0.05	0.11	0.23
DMI (kg/day)	1.03	1.14	1.05	1.02	0.06	0.53	0.42	0.22	< 0.01
DMI (% BW)	4.16	4.65	4.28	4.15	0.15	0.46	0.45	0.20	< 0.01
DMI (g DM BW^0.75^)	92.7	102.9	95.1	91.9	5.0	0.50	0.42	0.21	< 0.01
BCS^2^	2.45	2.41	2.50	2.39	0.05	0.21	0.75	0.53	0.09
FAMACHA	2.83	2.97	2.57	2.76	0.21	0.26	0.14	0.86	0.01
Gain (g/day)	91.1	77.7	74.2	70.0	9.8	0.41	0.28	0.66	-

^1^Time denotes Day effect for digestibility (DMD, OMD, NDFD, ADFD) and Week effect for DMI, BCS and FAMACHA. Two-way and three-way interaction including time effect are not included in table as *P* values > 0.16, except Week × Orange for BCS.

^2^Week × Orange = 0.05.

^3^O + L = Combination of Orange oil and Lespedeza hay.

The body condition score averaged 2.40 and no treatment effects were observed. FAMACHA averaged 2.8 and it was not affected by the supplementation of orange oil and lespedeza in diet; however, it varied throughout the weeks (*P* = 0.01), where at the beginning of the experiment averaged 2.8 and then decreased to 2.7 at the end of the experimental period. The inclusion of orange and lespedeza in diet did not affect average daily gain, which averaged 78.3 g/animal/day during the 5-wk period (Table 3). The conversion efficiency averaged 0.07 g BW produced/g DM (data not shown).

### Fecal Egg Count

**Figure 1.**
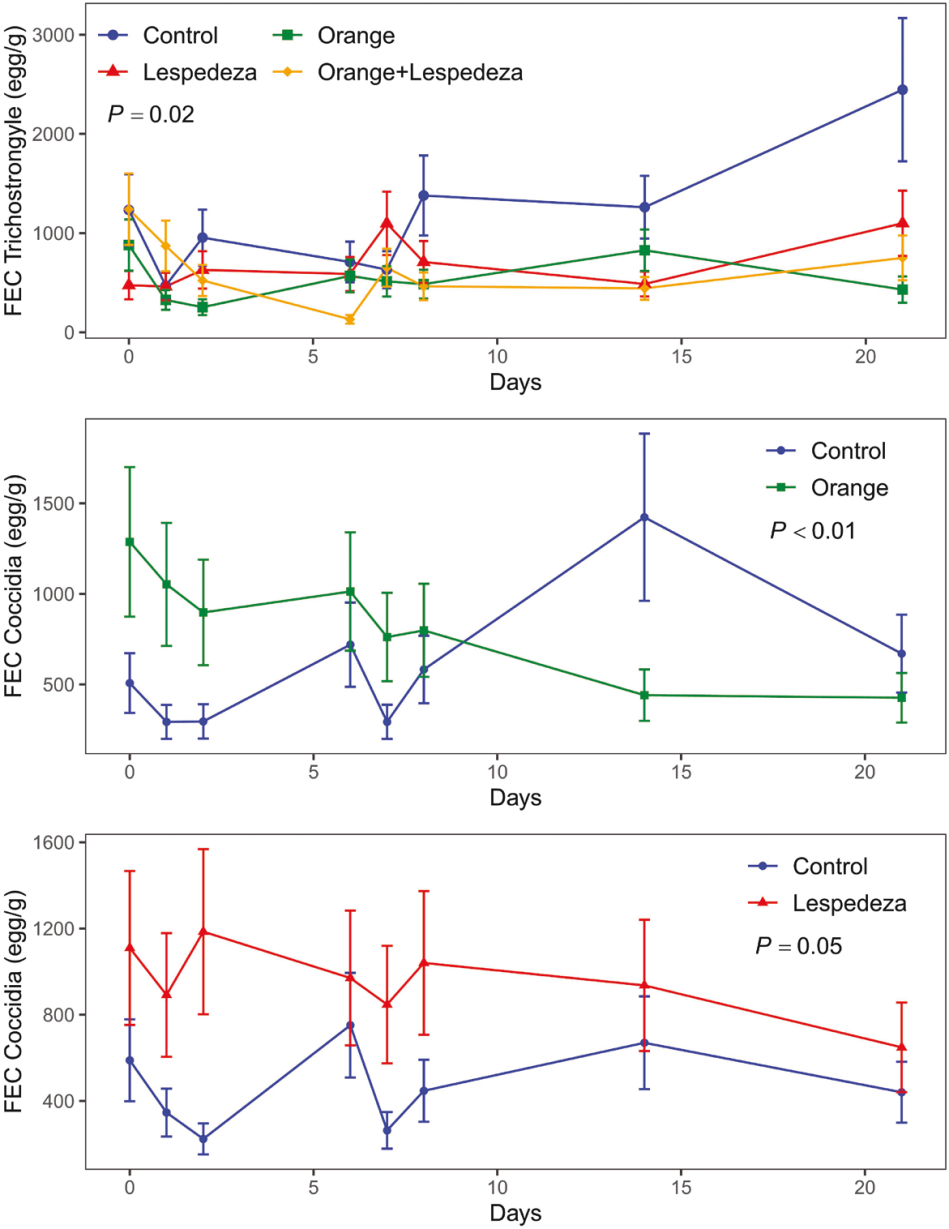
Effect of orange and lespedeza supplementation on fecal egg count (FEC; egg/g) of Trichostrongyle and Coccidia during the experimental period. Vertical bars denote the standard error. Bottom panels only show main effects because the orange × lespedeza × week interaction was not significant on affecting FEC coccidia at α = 0.10.

Including orange oil and lespedeza in the diet decreased FEC of Trichostrongyle but not of Strongyloide and Coccidia. On average, for the 21-d period, goats dosed with orange oil and/or lespedeza showed lower FEC than the control (Table 4). Additionally, while the FEC of Trichostrongyle in goats without orange oil and lespedeza increased from day 1 to day 21, dosing with orange oil and/or supplementing with lespedeza effectively maintained the FEC of Trichostrongyle similar for the 21-d period (~560 egg/g; *P* = 0.02; Figure 1). However, dosing with orange oil and/or feeding goats with lespedeza did not affect the FEC of Strongyloide, which averaged 166 egg/gram across treatments (Table 4).

**Table 4.** Treatment effects on Trichostrongylus, Strongyloide and Coccidia fecal egg count (FEC; egg/g) in response to Orange oil (O) administration and inclusion of Lespedeza hay (L) in the total mixed ration. *P* values only show the effect of orange oil, lespedeza, time, and the two-way interaction between orange oil and lespedeza (O × L)

	Treatments					*P* values			
	No Lespedeza		With Lespedeza						
FEC (egg/g)	Control	Orange	Lespedeza	O + L	SE	Orange	Lespedeza	O × L	Time
Trichostrongyle^1^	1006	495	654	541	90	<0.01	0.17	0.06	0.03
Strongyloide	55	277	118	211	193	0.36	0.83	0.66	0.02
Coccidia^2^^,^^3^	417	446	642	1361	227	0.20	0.04	0.28	0.06

^1^Orange × Lespedeza × Week = 0.02.

^2^Orange × Week < 0.01.

^3^Lespedeza × Week = 0.05.

Goats showed greater FEC of Coccidia when lespedeza was included in diet (*P* = 0.04; Table 4). This lack of control of Coccidia by lespedeza was only found during the first 2 wk of the 21-d period (*P* = 0.05; Figure 1), while by day 14 and 21 it did not differ with the ‘control’ treatment. Similarly, goats dosed with orange oil had greater Coccidia FEC compared with the control during the first days, a trend that was reversed during days 14 and 21, where orange oil reduced Coccidia and maintained FEC below 500 egg/g (*P* < 0.01; Figure 1).

### Blood Parameters

**Figure 2.**
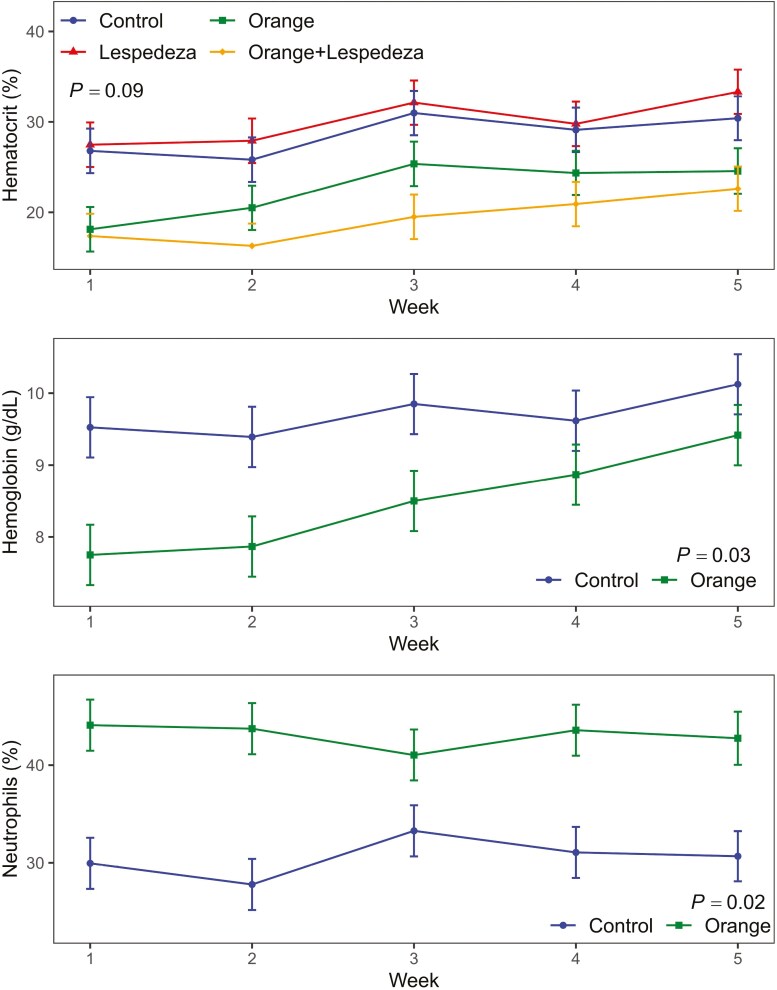
Effect of orange and lespedeza supplementation on hemoglobin (g/dL), hematocrit (%), and neutrophils (%) during the experimental period. Vertical bars denote the standard error. Bottom panels only show main effects because the orange × lespedeza × week and lespedeza × week interactions were not significant on affecting Hemoglobin (g/dL) and Neutrophils (%) at α = 0.10.

In general, there was no effect of lespedeza or interaction with orange oil on blood parameters (Table 5). Dosing with orange oil decreased hemoglobin concentration, hematocrit, and lymphocytes while increasing neutrophils (Table 5). The hemoglobin concentration of goats dosed with orange oil was lower than the control during the first 3 wk, and then increased and did not differ with the ‘control’ (Figure 2). Hematocrit in blood (%) increased from week 1 to week 5 and differed between treatments along the 5-wk period (*P* = 0.06). Goats dosed with orange oil and supplemented with lespedeza showed lower hematocrit concentration in blood, while goats under control or only fed with lespedeza showed higher concentrations of hematocrit in blood. Neutrophils concentration in blood tended to be stable during the 5-wk period; however, goats dosed with orange oil showed higher concentration of neutrophils (%) in blood compared with goats without orange oil (*P* = 0.02). Lastly, reticulocytes, monocytes, and basophils varied throughout the 5-wk experimental period; reticulocytes peaked at week 2 and then decreased, and while monocytes decreased from week 1 to week 5, basophils increased their concentration from week 1 to week 5.

**Table 5.** Treatment effects on blood parameters in response to Orange oil administration (O) and inclusion of Lespedeza hay (L) in the total mixed ration. *P* values only show the effect of orange oil, lespedeza, time, and the two-way interaction between orange oil and lespedeza (O × L)

	Treatments								
	No Lespedeza		With Lespedeza				*P* values		
Blood parameters	Control	Orange	Lespedeza	O + L	SE	Orange	Lespedeza	O × L	Time
Hemoglobin^1^ (g/Dl)	9.4	8.7	10.0	8.2	0.5	0.03	0.87	0.23	<0.01
Hematocrit^2^ (%)	28.6	22.8	30.1	19.3	2.4	0.01	0.69	0.31	<0.01
Reticulocytes^3^ (%)	2.7	2.4	2.5	2.2	0.6	0.59	0.71	0.89	0.05
Monocytes (%)	7.2	6.6	7.6	6.0	1.3	0.33	0.91	0.63	<0.01
Neutrophils (%)	32.1	41.5	29.3	43.9	2.9	<0.01	0.92	0.24	0.67
Lymphocytes (%)	58.7	49.0	61.7	48.9	2.1	<0.01	0.48	0.46	0.51
Eosinophil (%)	1.7	2.3	1.0	0.9	0.8	0.81	0.26	0.71	0.62
Basophils (%)	0.25	0.49	0.40	0.25	0.20	0.77	0.80	0.28	0.04

^1^Orange × Week = 0.03.

^2^Lespedeza × Week = 0.09.

^3^Orange × Lespedeza × Week = 0.06.

## DISCUSSION

Using natural alternatives to control GIN infestations in sheep and goats and reducing reliance on synthetic dewormers is crucial for sustainable small ruminant production systems. Orange oil has been shown to reduce Trichostrongylidae egg counts and total FEC in sheep infected with *Haemonchus contortus* with a single emulsion treatment through a 35-d experimental period (Carmona-Flores et al., 2024) . The efficacy of orange oil may be attributed to its high concentration (~95%) of d-limonene ( Squires et al., 2010), a lipophilic terpene with anthelmintic properties. Although the exact mechanism of action of d-limonene is not fully understood, its anthelmintic effects may result from its inhibitory impact on enzymes or plasma membrane pumps, which disrupts metabolic pathways and hinders parasite growth (Chagas et al., 2018) . The effectiveness of orange oil against Trichostrongylidae parasites aligns with previous research, though the inhibition rate in this study was 34.3%, compared to the 98% reported by Squires et al. (2010). Similarly, da Silva et al. (2021) observed a 96% and 77% reduction in fecal egg counts with two doses of 350 mg/kg orange oil on days 3 and 14, respectively.

Lower efficacy (~38%) was also observed in a previous trial with sheep (Carmona et al., 2024) following the same protocol, which could be related to the dose and frequency of treatment compared to other works in the literature. For Da Silva et al. (2021), greater suppression of GIN was obtained with a split orange oil (ORG) dose of 700 mg/kg BW (350 mg/kg BW per dose, with a 6-hour interval), but there was no effect on fecal egg counts when dosing ORG at 200, 300 and 400 mg/kg BW. The dose used in this trial (600 mg/kg BW) was derived from the study by Squires et al. (2010). Furthermore, variations in the efficacy could also be linked to differences in ORG composition as the source, extraction method, and quality of raw materials influence the concentration of active compounds (Dosoky and Setzer, 2018) . Helal et al. (2020) highlighted the potential for synergistic effects when different essential oils are combined. In Squires’ et al. (2010) research, the ORG used was a blend of orange terpene oil and Valencia orange oil, while in our study, only terpene oil was utilized. The extended duration of the trial may have also influenced the efficacy of ORG, as previous studies were only limited to 2 wk (Squires et al., 2010; da Silva et al., 2021).

Lespedeza, particularly sericea lespedeza (*L. cuneata*), plays a vital role in reducing FEC in small ruminants due to its high condensed tannins content. Unlike many other CT-rich forages such as bird’s-foot trefoil and sainfoin, which are cool-season legumes poorly adapted to the region’s warm climate and acidic soils, sericea lespedeza thrives in the infertile, warm conditions of the southern U.S. Research has demonstrated its antiparasitic effects, with studies showing lower FEC in goats grazing sericea lespedeza compared to non-CT grass pastures (Min and Hart, 2003; Min et al., 2004). Additionally, feeding dried, ground sericea lespedeza hay has significantly reduced FEC compared to feeding bermudagrass hay, offering the added benefit of flexible storage and feeding timing (Shaik et al., 2004) . The adaptability of sericea lespedeza to the southern U.S. and its effectiveness as an anthelmintic forage make it an important tool for parasite management in small ruminant production systems.

However, supplementing bermudagrass hay with Lespedeza had no significant effect FEC in goats. This may be attributed to the relatively low inclusion rate of lespedeza in the diet (~ 9%), whereas previous research has shown that lespedeza is most effective at reducing GIN egg counts and parasitic worm numbers when included at higher levels in the diet. Terrill et al. (2009)  demonstrated that lespedeza inclusion rates between 50% to 75% significantly reduced GIN egg counts in goat feces and diminished adult worm populations in the abomasum. It is recommended that SL-based diets should not exceed 60% to avoid adverse effects on animal performance while still maintaining an effective condensed tannin level of up to 25 g/kg DM. Therefore, the low inclusion rate used in our study likely limited the antiparasitic potential of lespedeza.

Nutrient digestibility (DM, OM, and NDF) was reduced with the inclusion of lespedeza in the diet of small ruminants; however, no effects were observed on DMI. A previous meta-analysis by Méndez-Ortiz et al. (2018) indicated that while tannin-rich plants like SL do not affect DMI, they increase metabolic costs and crude protein intake, often leading to reductions in digestibility. Similar results were obtained by (Min and Solaiman, 2018) , who reported a 12% decrease in dry matter digestibility (DMD). This was attributed to excessive condensed tannins impairing nitrogen digestibility and reducing ammonia production in the rumen, which ultimately affects overall nutrient absorption and animal performance. In addition to the effects of condensed tannins, higher concentrations of ADF (24.7% vs. 20.9%) and ADL (6.8% vs. 4.4%) with the inclusion of lespedeza in the diet could have affected nutrient digestibility as both ADF and ADL are structural components of the plant cell wall that are less digestible.

Despite observed differences in fecal egg counts, the absence of treatment effects on growth performance could be due to the relatively short study duration (5 wk) or the resilience of the experimental animals. Paolini et al. (2004) defined resilience as the animals’ ability to offset the negative impacts of parasitism while maintaining productive parameters. The natural infection observed in this study may not have been severe enough to affect body condition score (BCS). This finding is consistent with previous research, which also found no correlation between fecal egg counts and BCS (Vatta et al., 2002 ).

We observed an increase in neutrophil counts over the 5-wk experimental period after the animals received orange oil. This result was unexpected, considering d-limonene is known for its anti-inflammatory properties. Neutrophils are markers of inflammation, and their elevated presence typically indicates an inflammatory response. Da Silva et al. (2021) observed adverse reactions in lambs following the administration of ORG, including ataxia and head shaking, which could suggest that, at higher doses or certain formulations, ORG can trigger inflammation rather than suppress it. We speculate that the greater neutrophils counts may be indicative of some level of inflammation in the animals despite its proposed anti-inflammatory effects. Future research should investigate different dosages and formulations to balance the anthelmintic benefits of OEO with its potential side effects.

## CONCLUSION

While ORG demonstrated some anthelmintic efficacy by reducing GIN fecal egg counts in goats, the reduction was moderate compared to other studies, potentially due to dosage (amount and frequency) and formulation differences. Most of the trials in the literature, including the present, are of relatively short duration. Although anthelmintic proprieties of essential oils have been confirmed in vitro, many confounding factors exist in in vivo studies that can affect the outcomes. Orange oil shows promising results but remains to be tested on a production scale. Additionally, including sericea lespedeza in the diet at a relatively low rate (~9% on a DM basis) did not significantly impact fecal egg counts. Potentially, higher inclusion rates can enhance the antiparasitic effect but could come at the cost of reducing nutrient digestibility and, thus, performance. In this trial, despite the increase in fiber and reduction in nutrient digestibility of the diet, which resulted in lower DMI, lespedeza had no effect on performance, likely because of the short duration. Furthermore, an unexpected increase in neutrophil counts following ORG administration suggests possible inflammatory responses, warranting further research to optimize dosages and formulations for effective parasite control without adverse side effects. Despite observed variations in fecal egg counts, no significant impact on growth performance was noted, possibly due to the resilience of the goats or the short study duration.
